# Research Progress of Functionalized Drug Delivery Nanosystems in Regulating Depression

**DOI:** 10.3390/ph18121858

**Published:** 2025-12-05

**Authors:** Leying Qiang, Yongquan Huo

**Affiliations:** Psychology College, Shaanxi Normal University, Xi’an 710062, China; hyq@snnu.edu.cn

**Keywords:** depression, drug delivery nanosystems, functional modification, targeted regulation, neuroplasticity

## Abstract

Depression, as a highly prevalent psychiatric disorder, has emerged as a global public health challenge. Its pathological mechanisms involve the cross-regulation of multiple pathways, including neurotransmitter imbalance, neuroinflammation, and oxidative stress. Conventional oral antidepressants are limited in clinical application due to low blood–brain barrier penetration, significant side effects, and restricted therapeutic response rates. In recent years, drug delivery nanosystems (DDNs) have achieved precise regulation and repair of the pathological processes underlying depression through various functional modification strategies, such as targeted conjugation, stimuli responsiveness, and biomimetic simulation. Future efforts should focus on promoting their clinical translation via multi-functional integration, optimization of intelligent response mechanisms, and interdisciplinary collaboration. This review systematically summarizes the major pathogenic pathways of depression, as well as the mechanisms of action and research progress of functionalized DDNs in alleviating depression by targeting and remodeling key pathogenic pathways. It provides theoretical and technical references for the precise treatment of depression and the development of precision medicine for psychiatric disorders.

## 1. Introduction

Depression is a psychiatric disorder characterized by persistent low mood, diminished interest, and cognitive impairment. Its incidence has been on the rise annually, with a global prevalence of 4.4%, making it a major global public health concern [1]. Beyond severely impairing patients’ daily life, work, study, and social functioning, depression also significantly increases the risk of suicide and disrupts fundamental human biological functions, imposing a heavy economic and psychological burden on families and society [2]. Oral pharmacotherapy remains the primary approach for antidepressant treatment. Among the commonly used medications in clinical practice are selective serotonin reuptake inhibitors (SSRIs), noradrenergic and specific serotonergic antidepressants (NaSSA), and serotonin-norepinephrine reuptake inhibitors (SNRIs), etc. [3]. Additionally, anti-inflammatory drugs targeting abnormally elevated inflammatory factors (such as non-steroidal anti-inflammatory drugs and cytokine inhibitors) have also been employed in antidepressant therapy [4]. Despite their ability to regulate neurotransmitter levels, these antidepressant and anti-inflammatory drugs are limited in clinical application due to issues including restricted penetration [5], severe side effects [6], and low therapeutic response rates [7], which have driven the pursuit of antidepressant therapeutic strategies to optimize treatment outcomes.

Understanding the pathogenesis of depression is a primary prerequisite for developing effective therapeutic strategies. Depression has complex etiologies; it is often accompanied by multiple characteristics at the molecular level, primarily involving imbalances of neurotransmitters, such as serotonin (5-HT), dopamine (DA), and norepinephrine (NE), as well as uncontrolled neuroinflammation and oxidative stress. Conversely, these also serve as target molecules for the targeted reversal of depression. Excitingly, advances in nanotechnology have opened new opportunities for depression treatment. Benefiting from their inherent characteristics, such as small size and drug-loading capacity, drug delivery nanosystems (DDNs) have potential advantages in crossing the blood–brain barrier (BBB) and delivering antidepressants to the brain [8]. However, they still face challenges, including poor targeting accuracy [9], uncontrollable drug release, and unclear regulation of molecular mechanisms [10].

**Figure 1:**
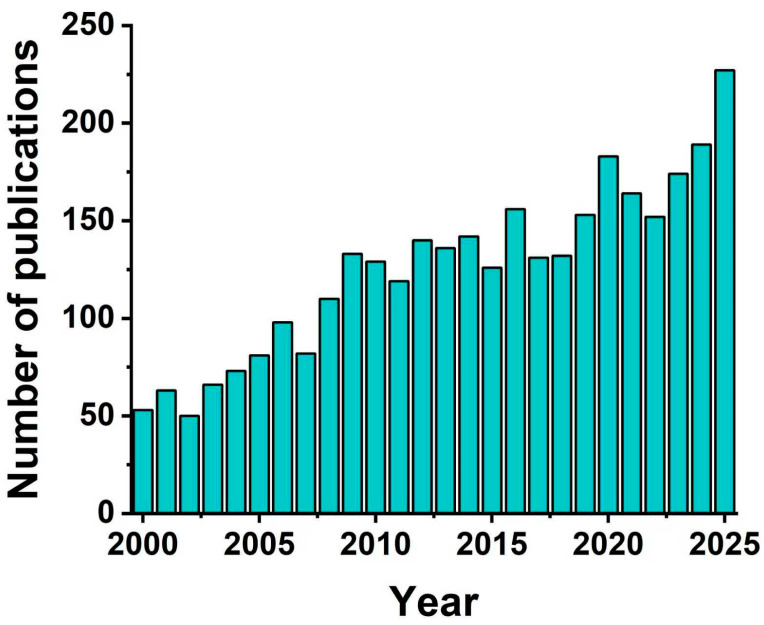
Trend chart of the number of SCI papers published on PubMed from 2000 to 2025 based on the search terms ‘drug delivery’ and ‘depression treatment’.

Fortunately, the construction of intelligent drug delivery systems via a series of functionalization strategies represents a potential breakthrough to address the aforementioned challenges. For instance: (1) attributing to the conjugation of the targeting molecules (e.g., transferrin) strategy, the constructed DDNs can specifically recognize pathological brain cells, precisely regulate the synthesis and metabolism of neurotransmitters, and ameliorate neurotransmitter imbalance [11,12]; (2) benefiting from the introduction of stimuli-responsive design strategy involving molecular switches (e.g., enzyme-responsive materials), the functionalized DDNs could release anti-inflammatory drugs or antioxidants in response to the special inflammatory microenvironments or oxidative stress sites as needed, thereby inhibiting neuroinflammation [13]; (3) with the help of incorporating reactive oxygen species (ROS)-scavenging strategy, nanoplatforms can normalize oxidative stress levels at damaged nerve sites and enhance the efficacy of antidepressant treatment [5], and the relevant molecular mechanisms would be clarified by the in vitro and in vivo studies of the functionalized DDNs. These diverse functionalized designs not only improve the precision and timeliness of treatment but also reduce systemic toxic side effects, holding great application potential in enhancing the efficacy of antidepressant therapy. Over the past 25 years, research interest and SCI publication in drug-delivery systems for depression have gradually increased (Figure 1). However, few studies have systematically reviewed the major pathogenic pathways of depression, the design strategies, development, and application advancements of the functionalized DDNs.

**Figure 2:**
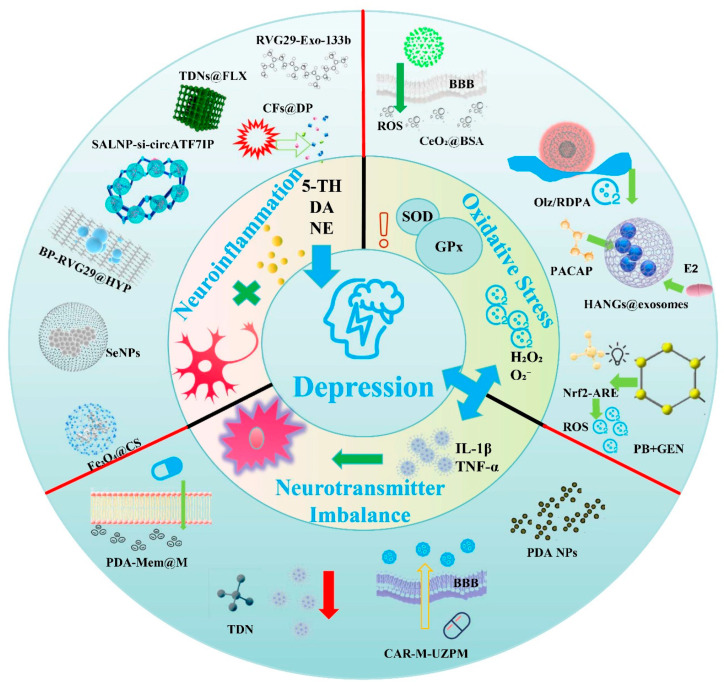
Various regulatory approaches to relieve depression based on functionalized DDNs are depicted in a schematic representation. These arrows represent the penetration of the BBB, the release of drugs and the regulation of target molecules for depression treatment.

The aim of this review is to systematically summarize the critical roles of major pathogenic pathways in the induction of depression, including neurotransmitter imbalance, neuroinflammation, and oxidative stress (Figure 2, Inner Ring). Furthermore, it will deeply illustrate the mechanisms underlying the construction of functionalized DDNs for targeted regulation of neurotransmitter levels, inhibition of neuroinflammation, and alleviation of oxidative stress (Figure 2, Outer Ring). Moreover, their research progress and technical advantages in antidepressant therapy are clearly evaluated. In addition, this review also objectively analyzes the current technical bottlenecks and translational challenges. This review not only provides theoretical support for the development of functionalized DDNs with antidepressant effects but also offers new directions for the precise regulation of antidepressant treatment. Therefore, it holds great significance for advancing the development of precision medicine in psychiatric disorders.

## 2. Pathogenic Pathways of Depression

### 2.1. Neurotransmitter Imbalance

Neurotransmitters represented by 5-HT, DA, and NE precisely regulate mood, cognition, and behavior; their imbalance is thus a prominent driver of depression. The pathogenic mechanisms mainly involve four pathways: abnormal neurotransmitter synthesis and metabolism, BBB dysfunction-mediated indirect imbalance, non-coding RNAs-dysregulated signaling, and BDNF downregulation with neuroplasticity impairment (Figure 2).

Firstly, abnormal synthesis and metabolism directly reduce 5-HT, DA, and NE levels in the synaptic cleft [14]. Tryptophan hydroxylase (TPH, a key 5-HT synthesis enzyme) and tyrosine hydroxylase (TH, critical for DA/NE synthesis) show impaired activity—TPH activity declines to reduce 5-HT precursors [15], while TH is inhibited by fluoride binding [16], ROS oxidative modification, or BDNF downregulation [17]. Additionally, overactivated monoamine oxidase (MAO) accelerates DA/NE degradation, and hyperfunctional presynaptic transporters (e.g., 5-HT transporter SERT) cause excessive neurotransmitter reuptake [18], disrupting the reward system and prefrontal cortex-limbic system signaling to induce low mood and anhedonia [19].

Secondly, BBB dysfunction amplifies neurotransmitter disorders. Inflammatory factors (e.g., tumor necrosis factor TNF-α, IL-6) and oxidative stress downregulate tight junction proteins (occludin, claudin-5), increasing barrier permeability and allowing peripheral toxins (e.g., fluoride) and inflammatory mediators to infiltrate the central nervous system (CNS) [20]. These substances directly inhibit the activity of neurotransmitter synthesis enzymes (e.g., pro-inflammatory factors block tryptophan to 5-HT conversion) and disrupt brain ionic homeostasis/enzymatic environments, thereby indirectly exacerbating 5-HT and DA metabolic disorders [21]. Typically, lipopolysaccharide (LPS)-induced inflammation increases the number of activated microglia (CD11b^+^CD45^+^) and their pro-inflammatory factor release, further suppressing neurotransmitter synthesis [22].

Thirdly, ncRNA-dysregulated signaling is a novel regulatory link to neurotransmitter imbalance. Circular RNA circATF7IP is significantly upregulated in MDD patients’ plasma, positively correlating with HAMD-24 scores [22]. Its overexpression activates inflammatory signaling, promotes microglial activation and pro-inflammatory factor (TNF-α, IL-6) release to indirectly inhibit neurotransmitter synthesis enzymes; it also sponges miRNAs or regulates epigenetics to disrupt presynaptic transporter (e.g., VMAT2) expression, impairing DA release and neurotransmitter transmission.

Finally, BDNF downregulation and neuroplasticity impairment convert transient neurotransmitter deficiency into persistent depressive symptoms. Chronic neurotransmitter deficiency inhibits the PI3K/AKT pathway and activates p38 MAPK, reducing BDNF expression [23]. Insufficient BDNF impairs neuronal trophic support, disrupts neurotransmitter synthesis enzyme regulation (e.g., TH) [24], inhibits neural precursor cell proliferation/differentiation (e.g., reduced hippocampal newborn neurons), decreases dendritic complexity and synaptic density, and activates the caspase-3 apoptotic pathway to accelerate mature neuronal death [25,26]. Ultimately, these changes result in hippocampal atrophy [27] and prefrontal cortex hypofunction [28], sustaining depression chronicity and lowering therapeutic response rates.

### 2.2. Neuroinflammation

Neuroinflammation disrupts CNS homeostasis through multi-dimensional mechanisms, characterized by a progression from immune activation to neural dysfunction. Its core pathogenic pathways include microglial abnormal activation, pro-inflammatory cytokine-mediated inflammation, epigenetic/autophagic dysregulation-driven inflammation, and inflammatory microenvironment response amplification.

Firstly, as the CNS’s innate immune cells, microglia initiate phenotypic transformation upon external stimulation to drive depressive pathology [29]. Peripheral inflammatory stimuli (e.g., LPS), chronic stress, or oxidative stress activate resting microglia to polarize into pro-inflammatory M1 phenotype [30]. M1 microglia induce depression by releasing pro-inflammatory cytokines interleukin-1β (IL-1β) and TNF-α to trigger local inflammation [31], and by excessively phagocytosing synapses and downregulating BDNF to impair synaptic plasticity, disrupting neuronal signaling [32].

Secondly, IL-1β and TNF-α are classic neuroinflammatory mediators in depression [33]. Released by activated microglia, astrocytes, or peripheral immune cells, these cytokines exacerbate pathology via inflammation amplification and neuronal damage [34]. They bind cell-surface receptors (e.g., TNF-α binding to TNFR1) to activate signaling pathways including nuclear factor κB (NF-κB) and p38 mitogen-activated protein kinase (p38 MAPK), promoting pro-inflammatory cytokine transcription in host and adjacent cells [35]. Additionally, they directly act on neurons: inhibiting TPH/TH activity to worsen neurotransmitter imbalance, and activating the HPA axis to sustain glucocorticoid elevation, impairing neuronal damage resistance [36].

Thirdly, epigenetic modifications (e.g., histone deacetylation) and autophagic dysfunction indirectly amplify microglial activation and inflammation by regulating gene expression and cellular homeostasis [37]. HDACs remove histone acetyl groups, condense chromatin, and relieve the inhibition of pro-inflammatory genes (e.g., TNF-α and IL-1β), thereby dysregulating inflammation [38]. Reduced autophagic flux (e.g., impaired autophagosome-lysosome fusion) leads to accumulation of damaged mitochondria and misfolded proteins, activating microglial DAMPs-recognition pathways (e.g., TLR4) [31,39], and it also decreases anti-inflammatory factors (e.g., BDNF) to weaken neuroprotection [40].

Finally, the inflammatory microenvironment amplification pathway involves region-specific abnormalities (decreased pH, elevated enzyme levels, ROS accumulation) that activate cytotoxic pathways [41]. Inflammatory cells (microglia, neutrophils) exhibit enhanced glycolysis, leading to lactic acid accumulation and acidification that activates neuronal ASIC1a, triggering calcium overload and apoptosis [42]. The increase in MMP-9 in the inflammatory microenvironment degrades tight junction proteins at the BBB [43], facilitating peripheral inflammatory factor infiltration to intensify inflammation, ultimately contributing to depression [44].

### 2.3. Oxidative Stress

As a key driver of depression pathology, oxidative stress disrupts intracellular redox homeostasis, progressing from excessive ROS generation to antioxidant system exhaustion and multi-dimensional neuronal damage via interconnected pathways. Its core pathogenic mechanisms include abnormal ROS generation, antioxidant defense exhaustion, oxidative stress-neuroinflammation crosstalk, neurotransmitter metabolism disorder, and neuroplasticity impairment.

Firstly, abnormal ROS generation stems from mitochondrial dysfunction and activated enzymatic reactions [44,45]. Chronic stress or inflammation disrupts neuronal mitochondrial respiratory chains, producing massive ROS (e.g., O_2_^−^, H_2_O_2_) [46], while abnormal activation of NADPH oxidase (NOX, especially neuron-specific NOX2) and MAO (releasing ROS during neurotransmitter degradation) further amplifies ROS accumulation [47]. Excessive ROS directly damages neuronal lipids, proteins, and DNA, and activates downstream inflammatory signaling pathways, leading to neuronal dysfunction and depressive-like behaviors.

Secondly, antioxidant defense system exhaustion arises from insufficient endogenous capacity. Endogenous antioxidants (SOD, GPx, GSH) show reduced activity or depleted levels due to persistent ROS attack or chronic stress [48,49,50], losing the ability to neutralize excess ROS. Meanwhile, abnormal activation of pro-oxidative enzymes (e.g., XO) [51] disrupts redox balance, exacerbating ROS toxicity and neuronal lipid peroxidation (evidenced by elevated malondialdehyde) [52]. Ultimately, this impairs neurotransmitter metabolism and synaptic plasticity, further contributing to depression progression.

Thirdly, oxidative stress and neuroinflammation interact synergistically via crosstalk. Generally, excessive ROS activates NF-κB [53,54] and p38 MAPK [55,56] pathways, promoting microglial activation and the release of pro-inflammatory cytokines, including IL-1β and TNF-α. These pro-inflammatory cytokines further activate NADPH oxidase and inducible nitric oxide synthase (iNOS) [57], exacerbating ROS generation and forming a vicious cycle [58]. Additionally, ROS and pro-inflammatory cytokines synergistically damage BBB tight junction proteins [59,60], facilitating peripheral inflammatory mediators infiltration to amplify neuronal damage and depressive behaviors.

Furthermore, oxidative stress disrupts neurotransmitter metabolism directly. Excessive ROS oxidatively modifies synthesis enzymes (TH, TPH) by targeting thiol groups, reducing their activity, and lowering DA/5-HT synthesis [61,62,63]. It also induces neuronal membrane lipid peroxidation, disrupting the conformation of 5-HT transporters and DAT, leading to abnormal neurotransmitter reuptake in the synaptic cleft [64,65], exacerbating neurotransmitter imbalance and triggering low mood and anhedonia.

Finally, oxidative stress impairs neuroplasticity to sustain depression chronicity. Excessive ROS inhibits neurogenesis and damages synaptic structures [66,67]: it causes oxidative DNA damage in neural precursor cells and downregulates BDNF, inhibiting hippocampal neural precursor cell proliferation/differentiation [66,68]; it also attacks synapse-associated proteins (e.g., Synapsin I, PSD95), reducing dendritic spine density and complexity to impair synaptic plasticity [68,69]. These changes induce hippocampal atrophy and cognitive impairment, perpetuating chronic depression. The mechanistic characteristics of the main pathogenic pathways of depression described above are summarized in Table 1.

**Table 1:** The main pathogenic mechanisms of depression.

Pathogenic Pathways of Depression	Main Pathways of Depression	Representative Signaling Factors	References
Neurotransmitter Imbalance	Neurotransmitter synthesis/metabolism abnormality	5-HT, DA, NE, TPH, TH, MAO, SERT	[14,15,16,17,18,19]
	BBB dysfunction-mediated imbalance	Occludin, Claudin-5, TNF-α, IL-6, LPS	[20,21,22]
	Non-coding RNA abnormal regulation	circATF7IP, TNF-α, IL-6, VMAT2	[22]
	BDNF downregulation and neuroplasticity impairment	BDNF, PI3K/AKT, p38 MAPK, Caspase-3	[23,24,25,26,27,28]
Neuroinflammation	Microglia abnormal activation	M1-type microglia, LPS, IL-1β, TNF-α	[29,30,31,32]
	Pro-inflammatory cytokine-mediated inflammation	IL-1β, TNF-α, NF-κB, p38 MAPK, HPA axis	[33,34,35,36]
	Epigenetic/autophagic dysfunction-mediated inflammation	HDACs, DAMPs, TLR4, BDNF	[37,38,39,40]
	Inflammatory microenvironment amplification	ASIC1a, MMP-9, ROS, Neutrophils	[41,42,43,44]
Oxidative Stress	Abnormal ROS generation	ROS, NOX2, MAO, Mitochondrial respiratory chain	[44,45,46,47]
	Antioxidant defense system exhaustion	SOD, GPx, GSH, XO, Malondialdehyde	[48,49,50,51,52]
	Oxidative stress-neuroinflammation crosstalk	ROS, NF-κB, p38 MAPK, IL-1β, TNF-α	[53,54,55,56,57,58,59,60]
	Oxidative stress-mediated neurotransmitter disorder	ROS, TH, TPH, 5-HT transporter, DAT	[61,62,63,64,65]
	Oxidative stress-mediated neuroplasticity impairment	ROS, BDNF, Synapsin I, PSD95	[66,67,68,69]

## 3. Regulation Strategies for Depression Therapy Based on Functionalized DDNs

Dysregulation of neurotransmitters, neuroinflammation, and oxidative stress induce aberrant alterations in the brain microenvironment. Drug therapy is known as a standard treatment option; however, conventional pharmacological therapies face inherent limitations, including poor water solubility, low delivery efficiency, and lack of target specificity. In contrast, nanoscale drug delivery systems (DDNs) inherently possess the advantages of enhancing drug water solubility and improving bioavailability, thereby serving as promising tools for the targeted therapy of depression. Co-controlled release remains a critical challenge. Considering that the pathological microenvironment of depression can produce some stimuli different from the normal environment, such as ROS, this serves as a good trigger for controlled release. Therefore, the construction of functionalized drug delivery systems exhibits substantial feasibility for depression therapy. Emerging studies have developed various functionalized nanosystems to specifically modulate neurotransmitter imbalance, neuroinflammation, and oxidative stress, achieving favorable therapeutic outcomes in depression treatment. These regulatory strategies primarily rely on the following three pathways, which have been extensively investigated with remarkable research progress.

### 3.1. Regulation Strategies of Neurotransmitter Imbalance in Depression by Functionalized DDNs

Given the critical role of neurotransmitter molecules in depression, they have become therapeutic targets for depression treatment. Therefore, the targeted regulation of neurotransmitter levels via the design and application of the functionalized DDNs has emerged as a powerful strategy to alleviate depression, with potential clinical application advantages.

**Figure 3:**
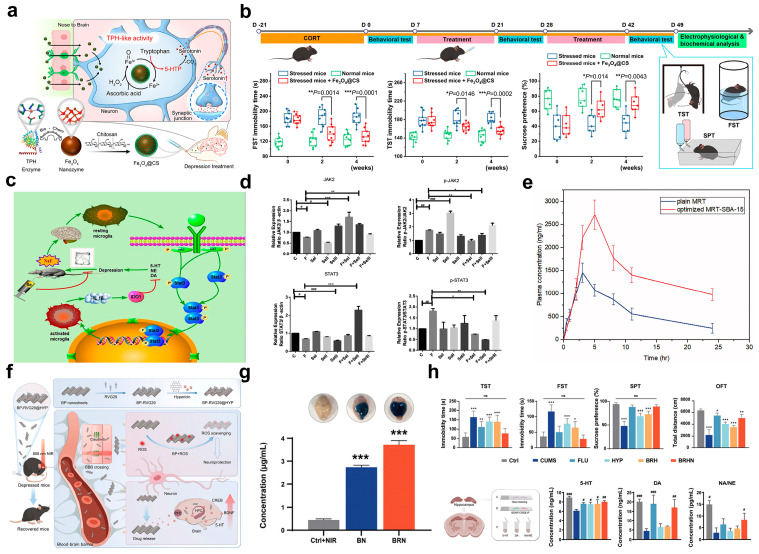
(**a**) Schematic diagram of restoring serotonin synthesis in neurons by a TPH-like DDNs for depression therapy. (**b**) Schematic timeline of anti-depression therapy, and depressive-like behavior was evaluated based on the FST, TST, and SPT. (**c**) Molecular mechanism diagram of anti-depression therapy via selenium-based DDNs. (**d**) Levels of mRNA expression of the JAK2-STAT3 signaling pathway and cytokine genes. (**e**) Pharmacokinetic parameters of MRT oral suspension and MRT-SBA-15-based DDNs. (**f**) Schematic diagram of the BRN DDNs for the treatment of depression. (**g**) Images and quantitative analysis of Evans Blue extravasation, an indicator of BBB permeability. (**h**) Depressive-like behavior was evaluated based on the FST, TST, SPT, OFT, and a schematic diagram of the antidepressant experiments, along with the levels of 5-HT, DE, and NA/NE in brain tissue. (**a**,**b**) Printed with permission from American Chemical Society [61]. (**c**,**d**) Printed with permission from American Chemical Society [16]. (**e**) Printed with permission from Informa UK Limited [71]. (**f**–**h**) Printed with permission from Wiley-VCH [12]. * *p* < 0.05, ** *p* < 0.01, and *** *p* < 0.001; # *p* < 0.05, ## *p* < 0.01, and ### *p* < 0.001, as compared with control group.

Targeting the pathway of abnormal neurotransmitter synthesis and metabolism, functionalized nanosystems can achieve precise regulation through two core strategies: enzyme-mimetic activity compensation and pathway inhibition. Relevant studies have confirmed their effectiveness in restoring neurotransmitter levels and improving depressive phenotypes. Specifically, in the direction of enzyme-mimetic activity compensation, Fang et al. were the first to report that Fe_3_O_4_ nanoparticles possess tryptophan hydroxylase-like activity (Figure 3a). They successfully verified that this property can restore serotonin (5-hydroxytryptamine) synthesis in the brain, thereby exerting an antidepressant effect [61]. To further enhance biocompatibility and intracerebral delivery efficiency, the research team modified the surface of Fe_3_O_4_ nanoparticles with chitosan (CS) to construct the Fe_3_O_4_@CS nanosystem, which can be directly delivered to the brain via the intranasal administration route [61]. Its mechanism of action lies in the fact that Fe_3_O_4_@CS can specifically catalyze the conversion of tryptophan to 5-hydroxytryptophan (a precursor of 5-HT) in stressed neurons, with synergistic participation from high levels of endogenous ascorbic acid and hydrogen peroxide. This effectively compensates for the inactivated tryptophan hydroxylase activity in the brain. In vivo experimental results further confirmed that Fe_3_O_4_@CS treatment can significantly restore the levels of 5-hydroxytryptophan and serotonin in the brains of depressive mouse models, while improving neuronal signal transduction ability, ultimately alleviating depressive-like behaviors effectively (Figure 3b). This research has also received support from other teams. For instance, Zhang et al. pointed out that the inactivation of tryptophan hydroxylase and the consequent reduction in 5-hydroxytryptamine levels are key links in the pathological mechanism of depression. They emphasized that using nanozymes to compensate for the inactivated function of this enzyme would be a feasible approach to restore serotonin levels and improve the pathological characteristics of depression [70]. In the direction of pathway-inhibition-based regulation, a study by Yang et al. provides a typical example (Figure 3c). They confirmed that the administration of selenium nanoparticles SeNPs at a dose of 1 mg/kg·d could precisely regulate the levels of monoamine neurotransmitters DA and NE, significantly alleviating depressive-like behaviors induced by 150 mg/L fluoride [16]. In-depth mechanistic studies revealed that fluoride exposure markedly activated the JAK2-STAT3 signaling pathway in the cortex, increasing the ratios of phosphorylated JAK2 (p-JAK2) to total JAK2 and the ratio of phosphorylated STAT3 (p-STAT3) to total STAT3 by 2.3-fold and 1.8-fold, respectively (Figure 3d). This pathway activation directly led to a 28% decrease in DA concentration, disordered NE secretion, accompanied by excessive microglial activation and the release of the pro-inflammatory cytokine IL-1β. In contrast, SeNPs with a particle size of 50 nm could effectively cross the BBB. By inhibiting JAK2 phosphorylation and blocking STAT3 nuclear translocation (reducing nuclear p-STAT3 levels by 63%), SeNPs not only restored the activity of TH (a key enzyme for DA/NE synthesis), elevating DA concentration to 1.3-fold of the normal level, but also inhibited the function of NE transporters, restoring NE levels in the hippocampus to 92% of the normal level. Additionally, SeNPs exerted neuroprotective effects, increasing the number of surviving cortical neurons by 38% and reducing neuronal vacuolar degeneration by 52%. This study is the first to reveal the targeted repair mechanism of selenium nanoparticles in mitigating fluoride-induced neurotransmitter imbalance and to provide key experimental evidence for the development of JAK/STAT pathway-inhibiting antidepressant nanomedicines.

Leveraging the unique physicochemical properties of nanomaterials, functionalized DDNs could effectively intervene in depression by improving the bioavailability of water-insoluble antidepressants and enhancing BBB penetration. Briefly, these strategies improve the efficiency of neurotransmitter-related drugs by overcoming poor water solubility and crossing the BBB, thereby indirectly regulating neurotransmitter metabolism. For the improvement of bioavailability, Musallam et al. significantly enhanced the antidepressant efficacy of mirtazapine (MRT) by optimizing its loading process into mesoporous silica (MSNs) nanostructures [71]. As a tetracyclic antidepressant (BCS Class II), MRT faces the critical issue of poor water solubility. To address this, the study further employed a Box–Behnken design to systematically optimize three key parameters: silica type (including SBA-15, MCM-41, and aluminate-MCM-41), drug-to-silica ratio (33.33–66.66%), and loading procedure (incipient wetness method, solvent evaporation, and solvent impregnation). The optimal formulation was ultimately determined as follows: MRT loaded into SBA-15 via the incipient wetness method at a drug ratio of 33.33%. This formulation achieved a drug loading efficiency of 104.05%, improved the water solubility of MRT to 0.2 mg/mL, and enabled a 100% drug dissolution rate within 30 min. To verify its in vivo efficacy, a rabbit pharmacokinetic study showed that this MSN delivery system increased the oral bioavailability of MRT by 2.14-fold (Figure 3e). By enhancing the regulatory efficiency of norepinephrine and serotonergic neurotransmission, it more effectively ameliorated neurotransmitter imbalance. Furthermore, multiple characterization techniques, including gas adsorption manometry, scanning electron microscopy (SEM), Fourier-transform infrared spectroscopy (FT-IR), differential scanning calorimetry (DSC), and X-ray powder diffraction (XRPD), collectively confirmed that the mesoporous structure of MSNs can form an amorphous solid dispersion with the drug. In addition to improving solubility and bioavailability through the above functional DDNs, more fundamentally overcoming the BBB is another key approach to enhancing the efficacy of depression treatment. Typically, Tan et al. constructed a novel nanoplatform, BP-RVG29@HYP (BRH), to optimize the delivery of natural antidepressant components via crossing the BBB with high efficiency (Figure 3f) [12]. This platform uses black phosphorus nanosheets as carriers, which are surface-modified with the neuron-targeting peptide Rabies Virus Glycoprotein-29 (RVG29) and then loaded with HYP, ultimately forming a highly specific drug delivery system. Under 808 nm near-infrared (NIR) light irradiation, BRH can not only specifically recognize and penetrate the BBB but also effectively reach brain lesion areas (Figure 3g). In vivo experimental results further confirmed that BRH not only significantly alleviated depressive-like behaviors in mice but also improved neurological function across multiple dimensions by fine-tuning the synthesis and metabolism of neurotransmitters and reducing oxidative stress levels (Figure 3h). Additionally, this system exhibited good safety with minimal adverse effects, providing a new paradigm for the efficient brain-targeted delivery of natural antidepressant drugs.

**Figure 4:**
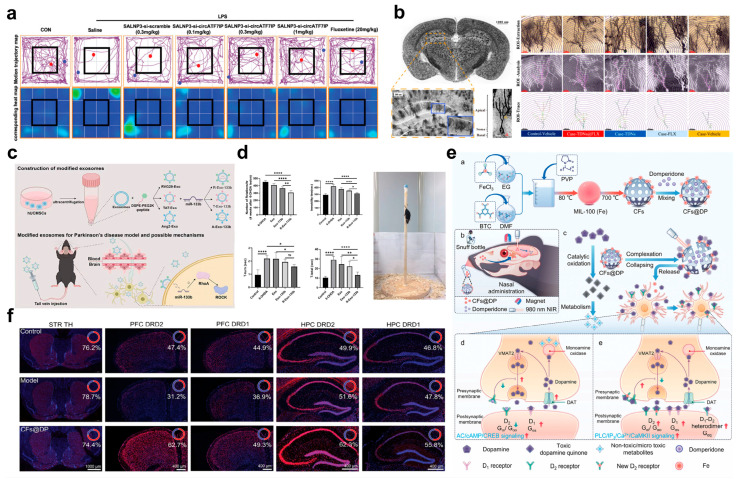
(**a**) Anti-depression therapeutic effects of SALNP3-si-circATF7IP, detected by the motion trajectory map and corresponding heat map of each group in the OFT. (**b**) TDNs@FLX DDNs induce early antidepressant effects by increasing dendritic complexity and dendritic spine density. The scale bar = 25 µm. (**c**) Schematic diagram of the construction of exosomes-modified DDNs and anti-depression therapy. (**d**) Effect of exosomes-modified DDNs on behavioral measures in vivo. (**e**) The synthesis of CFs@DP DDNs and its application in magnetic target-based drug delivery and neurotherapy. (**f**) Fluorescence staining of different brain regions. (**a**) Printed with permission from Wiley-VCH [22]. (**b**) Printed with permission from Elsevier [72]. (**c**,**d**) Printed with permission from American Chemical Society [73]. (**e**,**f**) Printed with permission from Wiley-VCH [74]. ns = not significant, * *p* < 0.05, ** *p* < 0.01, *** *p* < 0.001, **** *p* < 0.0001.

Beyond the aforementioned strategies, Ju et al. have also made significant progress in the field of non-coding RNA-targeted delivery. They first discovered that circular RNA circATF7IP is significantly upregulated in the plasma of patients with major depressive disorder (MDD), and its expression level is positively correlated with the 24-item Hamilton Depression Rating Scale (HAMD-24) scores. This finding reveals a close association between circATF7IP and the pathogenesis of MDD [22]. To address the challenge of low intracerebral delivery efficiency of non-viral vectors, the team further designed synergistic amine lipid nanoparticles SALNPs and achieved precise intranasal delivery of circATF7IP-targeting siRNA (si-circATF7IP) to the hippocampus of mice. In LPS-induced depressive mouse models, intranasal administration of SALNP-si-circATF7IP not only significantly reduced the number of CD11b^+^CD45^+^ activated microglial populations but also decreased the production of pro-inflammatory cytokines (TNF-α, IL-6), ultimately alleviating depressive-like behaviors in mice effectively (Figure 4a). This study not only confirms the potential of circATF7IP as a therapeutic target for MDD for the first time but, more importantly, the SALNP-mediated intranasal-brain siRNA delivery strategy provides a novel, efficient, and low-toxicity paradigm for circRNA-targeted depression therapy.

For the pathway of BDNF downregulation and neuroplasticity impairment, functionalized DDNs can intervene in the progression of depression by delivering neurotrophic factor modulators and promoting neurogenesis. Among relevant studies, Dou et al. proposed an innovative therapeutic strategy: they combined fluoxetine (FLX), a classic antidepressant, with tetrahedral DNA nanostructures (TDNs)–which possess both BBB penetration ability and neural stem cell proliferation-stimulating effects–to successfully synthesize a nanoscale complex TDNs@FLX [72]. To verify the antidepressant efficacy of this complex, the research team further established a chronic unpredictable stress (CUS)-induced depressive mouse model and systematically evaluated its therapeutic effects on various depressive symptom manifestations. Results showed that the TDNs@FLX complex exhibited excellent distribution in brain tissues. It not only effectively promoted the proliferation of neural precursor cells, but also significantly increased neuronal dendritic complexity and dendritic spine density (Figure 4b). These two effects work synergistically, ultimately enabling the complex to exert rapid and long-lasting antidepressant effects, thus providing a solution for neuroplasticity impairment associated with BDNF downregulation. Meanwhile, Jiang et al. developed a peptide-modified exosome-mediated precise DDNs. They modified the surface of natural exosomes with BBB-shuttling peptides (including three peptide segments: RVG29, TAT, and Ang2) and loaded miR-133b into them, ultimately constructing a nanodrug carrier with brain region-targeting capability (Figure 4c) [73]. Experimental results confirmed that among all modification schemes, RVG29-modified exosomes (RVG29-Exo-133b) showed the optimal performance: they not only exhibited excellent BBB penetration efficiency (with intracerebral uptake 3.7-fold higher than that of the unmodified group) but also demonstrated good biosafety. Further mechanistic studies revealed that this carrier could significantly reduce the phosphorylation level of Tau protein (with a 41% decrease in phosphorylation at the p-Tau Ser202/Thr205 site) by targeted regulation of the RhoA-ROCK signaling pathway. This, in turn, restores the expression of vesicular monoamine transporter 2 (VMAT2) on the presynaptic membrane of dopaminergic neurons, ultimately promoting the synthesis and release of DA [73]. To further verify its efficacy in pathological models, the research team tested it in a 6-hydroxydopamine (6-OHDA)-induced Parkinson’s disease (PD)-depression model. Results showed that this system could restore the DA concentration in the striatum to 89% of the normal level; meanwhile, by improving the neurotransmitter transmission efficiency of the nigrostriatal pathway, it not only increased the motor function score of mice (with a 2.3-fold extension in the rotarod test latency), but also alleviated depressive-like behaviors simultaneously (with a 38% reduction in the immobility time in the forced swim test). Using behavioral tests, immunohistochemistry, and other methods, this study comprehensively evaluated the efficacy of RVG29-Exo-133b in treating PD in mice, clearly confirming that this carrier can improve motor function, reduce depressive symptoms, enhance dopaminergic neuron function, and alleviate 6-OHDA-induced neural damage in PD mice (Figure 4d). It thus provides another important technical approach for neural function repair associated with BDNF downregulation. In addition to the two aforementioned strategies, Hu et al. also innovated from the perspective of “responsive release and receptor regulation” by developing a CFs@DP intranasal dual-responsive DDNs, aiming to achieve rapid brain-targeted delivery without addiction (Figure 4e), which consisted of carbonized MIL-100 (Fe) frameworks (CFs) and domperidone (DP). Under the dual stimulation of NIR photothermal effect and catecholamine complexation, CFs@DP can release iron ions and DP in a controlled manner, and these two released components exert synergistic effects [74]. On the one hand, they simultaneously upregulate the density of D1/D2 receptors in the prefrontal cortex (PFC) and hippocampus (HPC), thereby enhancing neurotransmitter signal transmission efficiency (Figure 4f); on the other hand, they activate the BDNF pathway, directly improving neuroplasticity. In vivo experiments further confirmed that continuous administration of the system to 50–60-day-old mice for 10 days significantly increased the density of D1/D2 receptors in brain regions and effectively alleviated depressive-like behaviors. This system not only solves the problem of slow synaptic plasticity regulation in traditional antidepressant therapy, but also combines safety and rapid efficacy, providing a novel idea for the intervention of BDNF downregulation pathways.

### 3.2. Regulation Strategies of Neuroinflammation in Depression by Functionalized DDNs

The development and progression of neuroinflammation in depression rely on the synergistic action of multiple pathways, including abnormal microglial activation, pro-inflammatory cytokine cascades, epigenetic and autophagic abnormalities, and amplification of the inflammatory microenvironment. Generally, the targeted intervention strategies mediated by functionalized DDNs have been developed for depression therapy based on the pathological characteristics of different pathways. These strategies not only achieve precise blocking of inflammatory signals but also enhance BBB penetration efficiency and biosafety by optimizing carrier properties.

**Figure 5:**
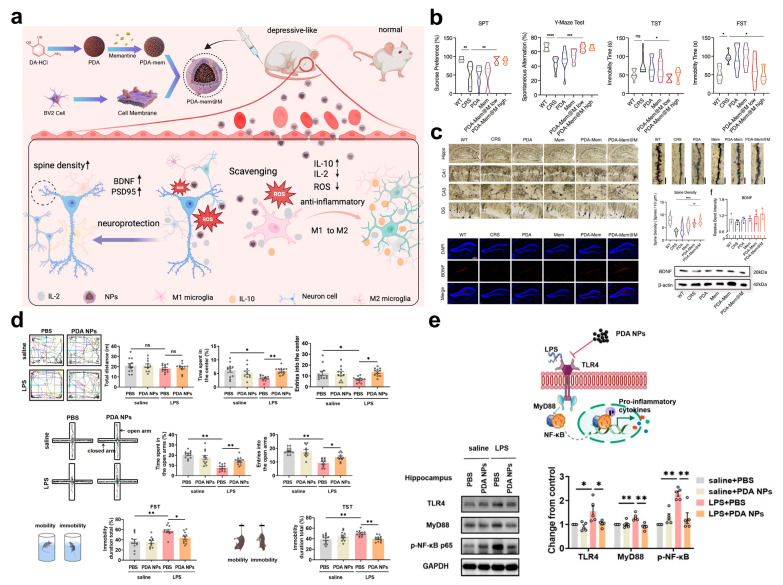
(**a**) Schematic illustration of the synthesis of PDA-Mem@M DDNs and regulation for neuroinflammation and neuroplasticity in depression. (**b**) PDA-Mem@M DDNs ameliorates depression-like behaviors of CRS mice. Scale bars: 100 µm for golgi-stained hippocampal neurons, 5 µm for golgi-stained dendrites, and 200 µm for immunofluorescent staining. (**c**) PDA-Mem@M DDNs reverse dysfunctional synaptic plasticity, as imaged by the Golgi-stained hippocampal neurons and dendrites with corresponding quantification, and BDNF expression in DG hippocampal neurons. (**d**) PDA-based DDNs improved anxiety- and depression-like behaviors evaluation. (**e**) Schematic mechanism of PDA-based DDNs restraining LPS-induced microglial activation and the corresponding Western blot analysis. (**a**–**c**) Printed with permission from Wiley-VCH [75]. (**d**,**e**) Printed with permission from Springer Nature [76]. Data are shown as mean ± SD. Statistical analysis was performed using one-way ANOVA with Dunnett’s test. ns = not significant, * *p* < 0.05, ** *p* < 0.01, *** *p* < 0.001, and **** *p* < 0.0001.

Targeting the abnormal activation of microglia, a core pathogenic pathway of neuroinflammation, researchers have achieved precise blocking of this pathway through biomimetic carrier design and multi-mechanism synergy. Among relevant studies, Jiang et al. constructed a microglia-biomimetic nanosystem, PDA-Mem@M, which uses polydopamine (PDA) as the core, is modified with memantine (Mem), and encapsulated with Mem of the microglial cell line BV2. Leveraging the biomimetic properties of the cell membrane, this nanosystem penetrates the BBB and targets activated microglia (Figure 5a) [75]. Within the system, PDA scavenges ROS via its catechol structure to attenuate the excessive activation signals of microglia; meanwhile, Mem, which is released in a pH-responsive manner, promotes the secretion of BDNF by activating the TrkB/BDNF pathway. These two components synergistically inhibit the release of pro-inflammatory cytokines (TNF-α, IL-1β) and induce the polarization of microglia toward the anti-inflammatory phenotype. Ultimately, in a chronic restraint stress (CRS)-induced depression model, the nanosystem significantly reduces the activation ratio of microglia in the hippocampus, ameliorates synaptic structural damage, and achieves superior antidepressant effects compared to monotherapy (Figure 5b,c). Similarly, Zhu et al. synthesized melanin-like polydopamine nanoparticles PDA NPs with a size of approximately 250 nm to intervene in microglial activation [76]. Rich in phenolic hydroxyl groups, PDA NPs exhibit excellent free radical scavenging ability, and their antioxidant activity has been confirmed to be significant in in vitro experiments. By establishing an LPS-induced inflammatory depression model in mice, it was found that intraperitoneal injection of PDA NPs significantly alleviated depressive-like behaviors in the mice (Figure 5d). Further mechanistic studies revealed that PDA NPs reduce peripheral and central inflammatory responses through multiple dimensions (Figure 5e), specifically manifested as inhibiting splenomegaly, decreasing the levels of serum inflammatory cytokines, suppressing excessive activation of microglia, and simultaneously promoting the repair of synaptic structures. Additionally, both in vitro and in vivo experiments have demonstrated that PDA NPs possess good biocompatibility, providing safety support for their subsequent applications.

**Figure 6:**
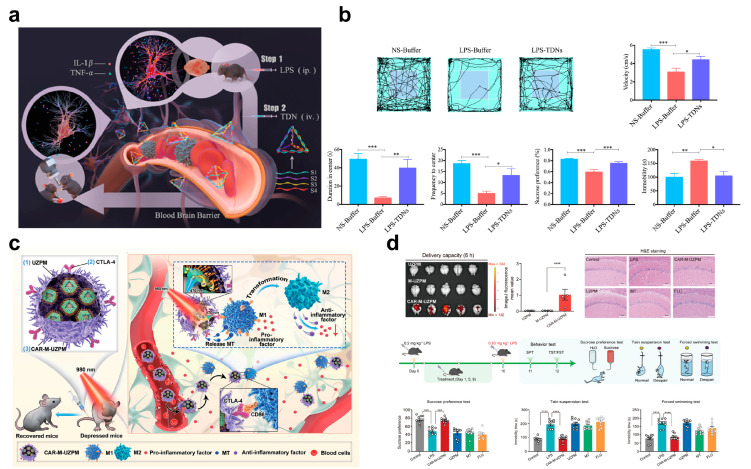
(**a**) Schematic illustration of the anti-depression effect of TDNs and DDNs on LPS-induced neuroinflammation in vivo. (**b**) Effect of TDNs on LPS-induced depression-like behavior. (**c**) The construction of the functionalized CAR-M-UZPM DDNs and the treatment of inflammation-related depression, (**d**) the effective penetration of crossing the BBB, and the antidepressant effect of CAR-M-UZPM DDNs; Scale bar = 100 µm. (**a**,**b**) Printed with permission from Elsevier [77]. (**c**,**d**) Printed with permission from Wiley-VCH [79]. * *p* < 0.05, ** *p* < 0.01, *** *p* < 0.001, **** *p* < 0.0001. Data are means ± S.E.M.

In the pro-inflammatory cytokine-mediated inflammatory cascade amplification pathway, the core intervention target is to reduce the levels of key factors such as IL-1β and TNF-α. The TDNs developed by Yang et al. achieve antidepressant effects by directly regulating the expression of these factors [77]. Study results showed that TDN intervention significantly increased movement speed, residence time in the central area, and entry frequency of LPS-treated mice in the OFT (Figure 6a). Meanwhile, it enhanced sucrose preference and shortened the immobility time in the TST. Improvements in these behavioral indicators directly reflect the alleviation of depressive-like symptoms. Further immunofluorescence assays revealed that peripheral administration of LPS activates the inflammatory response in mice and induces obvious depressive-like behaviors. In contrast, TDN can block signal transmission of the inflammatory cascade at the source by downregulating the expression levels of pro-inflammatory cytokines (e.g., IL-1β, TNF-α) in the brain. Ultimately, this effectively inhibits the inflammatory response and ameliorates depressive-like behaviors (Figure 6b).

For the inflammatory pathway mediated by abnormal epigenetic regulation and autophagy, researchers have focused their intervention targets on HDACs. They alleviate inflammation through a dual mechanism of regulating epigenetic modifications and autophagic function. In a study by Baek et al., a novel HDAC inhibitor, Compound 5, was developed. This compound significantly enhanced pan-HDAC inhibitory efficacy at the cellular level, providing a highly efficient molecular tool for pathway intervention [78]. Mechanistic studies revealed that Compound 5 can induce the initiation of basal autophagy in microglia while reducing the level of iNOS. As a result, it exerts anti-inflammatory and neuroprotective effects in both human and mouse cell lines, achieving a synergistic effect of “autophagy activation-inflammation inhibition”. Further in vivo experiments confirmed that this compound can alleviate inflammation-induced depressive symptoms in mice: by triggering a cascade reaction of “autophagy inhibition of nitric oxide production”, it effectively suppresses LPS-induced microglial activation. Moreover, by inhibiting the excessive activation of microglia in the mouse brain, it significantly ameliorates depressive-like behaviors. This study not only provides experimental evidence for targeting HDAC11 to promote synaptic regeneration and repair neural networks but also establishes the potential of HDAC11 as a novel therapeutic target for depression.

Targeting the inflammatory microenvironment response amplification pathway, environment-responsive nanosystems leverage the characteristics of the microenvironment to trigger carrier responses and achieve precise drug release, thereby enabling spatiotemporally controlled intervention within the pathway. Among such systems, the UZPM photo-responsive nanosystem developed by Liu et al. is highly representative. This nanosystem consists of up-conversion nanoparticles (UCNP@ZIF-8), photoacid (PA), and melatonin (MT). After being introduced into macrophages via functional liposome fusion, aldehyde-modified CTLA-4 is used as a chimeric antigen receptor (CAR) targeting moiety, which modifies the cell surface through hydroxylamine condensation to finally construct the CAR-M-UZPM DDNs (Figure 6c) [79]. This nanosystem can penetrate the BBB via a photo-responsive mechanism, specifically recognize M1-type activated microglia in the CNS, and inhibit their polarization while inducing a sustained vaccine-like anti-inflammatory effect. Both in vitro and in vivo experiments have confirmed that it can effectively block the occurrence and development of inflammation-related depression. This design not only utilizes the targeting characteristics of the inflammatory microenvironment but also combines photo-response to achieve spatiotemporal control of drug release, providing an innovative strategy with both BBB penetration and immunomodulatory functions for targeted neuroinflammation therapy (Figure 6d).

### 3.3. Regulation Strategies of Oxidative Stress in Depression by Functionalized DDNs

The core of the oxidative stress-related pathogenic pathway in depression lies in the imbalance of ROS. Specifically, excessive ROS generation leads to the exhaustion of the antioxidant system, which, in turn, causes neuronal damage through multidimensional pathological cascades, ultimately forming multiple interconnected pathological pathways. Correspondingly, functionalized DDNs were constructed to target core targets of different pathways and achieve precise intervention in oxidative stress through differentiated modifications, such as stimulus responsiveness, precise targeting, and biomimetic simulation.

**Figure 7:**
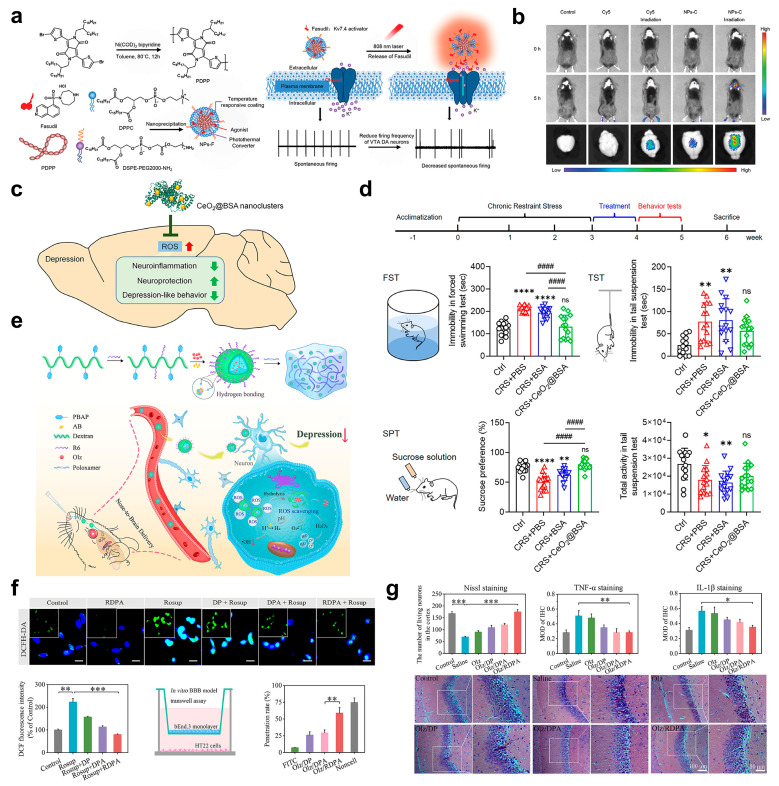
(**a**) Schematic illustration of the preparation of NPs-F DDNs and photothermal modulation of depression. (**b**) In vivo BBB permeability imaging of DDNs. (**c**) Schematic illustration of the CeO_2_@BSA DDNs for depression therapy via ROS scavenging. Arrows represent an increase or decrease in factor levels or treatment effects. (**d**) CeO_2_@BSA DDNs ameliorate depression-like behaviors of CRS mice. (**e**) Schematic diagram of nasal-brain administration of Olz/RDPA DDNs for depression therapy. (**f**) ROS depletion analysis and BBB penetration evaluation of DDNs. (**g**) The antidepressant effect in vivo of DDNs, characterized by neuronal injury analysis, expression of inflammatory factors, and morphological changes in hippocampal neurons. (**a**,**b**) Printed with permission from Wiley-VCH [80]. (**c**,**d**) Printed with permission from the American Chemical Society [81]. (**e**–**g**) Printed with permission from Elsevier [82]. Scale bars: no bar for (**b**), 10 µm for (**f**), and 100 µm or 50 µm for (**g**). ns = not significant, * *p* < 0.05, ** *p* < 0.01, *** *p* < 0.001, and **** *p* < 0.0001; and #### *p* < 0.0001.

Targeting the pathway of abnormal ROS generation, functionalized DDNs primarily achieve precise blocking of the pathway through the complementary effect of “photoresponse-triggered release” and “targeted ROS scavenging”. Among these nanosystems, the NIR-responsive nanosystem developed by Li et al. innovatively integrates the functions of “photothermal conversion-ROS sensing-sequential drug release” (Figure 7a). On the one hand, this nanosystem leverages the deep-tissue penetration property of NIR light and the photothermal conversion capability of nanoparticles to efficiently penetrate the BBB (Figure 7b) [80]. On the other hand, it loads fasudil, a Kv7.4 potassium channel activator, into an NIR photosensitive carrier. Triggered by NIR light irradiation, the nanosystem can dynamically adjust the drug release rate in response to changes in ROS concentration in the oxidative stress microenvironment. This specifically regulates the firing frequency of dopaminergic neurons in the ventral tegmental area (VTA), thereby inhibiting ROS-induced excessive neuronal excitation and blocking ROS-mediated damage to neural function at the “downstream effect” level. Furthermore, the CeO_2_@BSA nanoclusters (with a size of only 2 nm) synthesized by Fu et al. directly scavenge excessive ROS by virtue of dual enzymatic activities similar to SOD and catalase (Figure 7c). Their ultra-small size significantly enhances BBB penetration efficiency. Notably, modification with bovine serum albumin (BSA) not only improves biocompatibility but also accelerates in vivo metabolism rate, effectively overcoming the drawbacks of conventional antioxidants, such as “low antioxidant efficiency, poor structural stability, and difficulty in in vivo clearance”. Further in vitro and in vivo experiments confirmed that these nanoclusters can efficiently cross the BBB, precisely target oxidative stress lesion areas to scavenge ROS, and simultaneously avoid systemic adverse effects, thereby reducing ROS accumulation at the “source” and inducing the good anti-depression effect (Figure 7d) [81].

Targeting the antioxidant defense system exhaustion pathway, nanosystems rebuild the intracerebral redox homeostasis through a dual strategy of “drug-loading supplementation” and “avoiding depletion of the endogenous system”. The Olz/RDPA nanoparticles designed by Liu et al. follow this logic: this system co-loads olanzapine (Olz), an antidepressant, and ammonia borane (AB), an H_2_ donor, into “ROS-responsive dextran (DEX) derivatives modified with hexa-arginine (R6)” and disperses them in a poloxamer-based thermo-responsive hydrogel (Figure 7e) [82]. During delivery, the intranasal-brain pathway ensures efficient drug entry into the brain; the thermo-responsive property of the hydrogel enables local drug retention in the nasal cavity to prolong the action time; and the R6 modification enhances targeting by binding to receptors on the neuron surface. When the nanoparticles effectively cross the BBB and reach the oxidative stress lesion area, the ROS-responsive dextran backbone dissociates as local ROS levels increase (Figure 7f). The released AB assists in ROS scavenging by providing H_2_, while Olz specifically inhibits the functional abnormality of the 5-HT transporter induced by oxidative stress. Meanwhile, the sustained drug-release properties of the hydrogel carrier continuously supplement antioxidant substances, avoiding excessive consumption of endogenous antioxidants such as GSH. Both in vitro and in vivo experiments confirmed that, through the synergistic effect of “ROS scavenging + neurotransmitter protection”, this nanosystem can effectively alleviate depressive-like behaviors (Figure 7g) [82].

**Figure 8:**
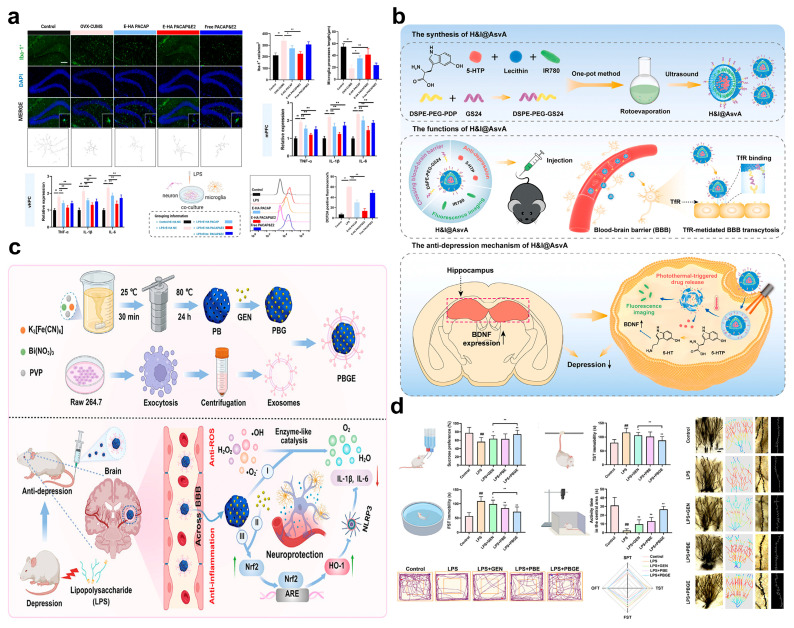
(**a**) The effect of HA NGs@exosomes on oxidative stress and microglial activation. Scale bar: 100 µm. (**b**) Schematic illustration of H&I@AsvA synthesis and its antidepressant mechanism. (**c**) Schematic illustration for the construction of PBGE DDNs and their inflammatory depression therapy. (**d**) PBGE DDNs ameliorate depression-like behaviors and their neuroplasticity effect. (**a**) Printed with permission from Springer Nature [84]. (**b**) Printed with permission from Elsevier [85]. (**c**,**d**) Printed with permission from Wiley-VCH [86]. ## *p* < 0.01; * *p* < 0.05, ** *p* < 0.01. Data were means ± SD.

Targeting the oxidative stress-neuroinflammation crosstalk pathway, nanosystems break the vicious cycle of “increased ROS-inflammatory activation-further increased ROS” through dual-dimensional intervention of “ROS scavenging” and “inflammation inhibition”. A study by Yu et al. provides target-based evidence for this strategy: they found that the expression of PARP14 in the hippocampus of mice with CUS was significantly increased. PARP14 can positively regulate nicotinamide nucleotide transhydrogenase (NNT) in microglia, thereby enhancing ROS scavenging capacity; if NNT is deficient, ROS accumulation will drive the polarization of microglia toward a pro-inflammatory phenotype [83]. The nanodrug developed based on this finding can precisely intervene in the oxidative stress state of hippocampal microglia by targeted delivery of PARP14 activators or NNT potentiators. It not only enhances endogenous ROS scavenging by upregulating NNT activity but also reduces the release of pro-inflammatory cytokines such as IL-1β and TNF-α by inhibiting microglial activation. While blocking the “ROS-inflammation” crosstalk pathway, it avoids the risk of systemic immunosuppression. Similarly, the exosome membrane-wrapped hyaluronic acid nanogels (HA NGs@exosomes) designed by Hu et al. achieve synergistic intervention by loading pituitary adenylate cyclase-activating polypeptide (PACAP) and estrogen (E2) [84]. The natural biomimetic properties of the exosome membrane endow the system with efficient BBB-crossing ability and low immunogenicity. After ROS-responsive modification, the hyaluronic acid backbone can trigger drug release in the cerebral microenvironment under oxidative stress. On the one hand, released PACAP and E2 directly neutralize ROS through the antioxidant components carried by the exosome membrane; on the other hand, they activate the endogenous antioxidant system (e.g., upregulating the activities of SOD and GPx) while inhibiting excessive microglial activation. This blocks the crosstalk pathway from the dual dimensions of “ROS scavenging” and “inflammation inhibition” (Figure 8a). In a mouse model of perimenopausal depression, intranasal administration of this system rapidly improved behavioral indicators, directly confirming its intervention effect on the crosstalk pathway [84].

Targeting the oxidative stress-mediated neurotransmitter metabolism disorder pathway, nanosystems achieve intervention through a combined strategy of “targeted delivery of neurotransmitter precursors + protection of enzyme activity”. A typical representative is the NIR light-responsive artificial synaptic vesicles developed by Chang et al.: 5-hydroxytryptophan (5-HTP), the payload of this system, is the direct precursor of 5-HT (Figure 8b) [85]. It can bypass the bottleneck of “reduced tryptophan hydroxylase activity caused by ROS-induced oxidative modification” and directly provide raw materials for 5-HT synthesis. Meanwhile, the ROS-responsive release property of the carrier ensures precise accumulation of 5-HTP in oxidative stress lesion areas, avoiding the oxidative degradation of 5-HTP by ROS. In addition, the NIR photothermal effect can transiently increase the permeability of the BBB; when combined with the intranasal-brain delivery route (direct entry into the brain via the nasal olfactory mucosa-olfactory bulb), this significantly enhances the targeted delivery efficiency of the drug into the brain. Ultimately, by supplementing the level of 5-HT in the brain, the system effectively ameliorates the neurotransmitter metabolism disorder mediated by oxidative stress, fully embodying the regulatory logic of “targeted supplementation -environmental protection” of nanosystems for this pathway [85].

Targeting the oxidative stress-mediated neuroplasticity impairment pathway, nanosystems achieve intervention through a synergistic mechanism of “BDNF pathway activation-neuroprotection”. A typical example of this design is the Prussian blue (PB) nanotherapeutic system developed by Wang et al.: This system uses porous PB as the core carrier, which is self-loaded with teniposide (GEN) and modified with exosome functionalization to enhance BBB permeability (Figure 8c) [86]. Among its components, PB, with its unique multi-enzyme mimetic activities (SOD-like, CAT-like, and peroxidase-like activities), can efficiently scavenge ROS in lesion areas while creating a slightly acidic environment to provide antioxidant protection for GEN. After the system crosses the BBB and reaches the brain, PB and GEN synergistically activate the Nrf2-ARE pathway. As a core transcription factor for endogenous antioxidant and anti-inflammatory responses, the activation of Nrf2 not only induces the expression of antioxidant proteins such as heme oxygenase-1 (HO-1) and NAD(P)H: quinone oxidoreductase 1 (NQO1) but also promotes the proliferation of neural precursor cells by upregulating the expression of BDNF. Meanwhile, ROS scavenging by PB reduces oxidative DNA damage in neural precursor cells, and GEN protects mature neurons and synaptic structures by inhibiting apoptotic pathways such as caspase-3. Ultimately, through the synergistic effects of “ROS scavenging-signal activation-neuroprotection”, this nanosystem repairs oxidative stress-mediated neuroplasticity impairment, providing a new approach for the intervention of depression chronicization (Figure 8d) [86] (Table 2).

## 4. Discussion and Future Perspectives

This review systematically summarizes the molecular mechanisms and pathological characteristics of three core pathogenic pathways in depression: neurotransmitter imbalance, neuroinflammation, and oxidative stress, and analyzes the intervention strategies of functionalized nanoparticle drug delivery systems. These pathways form a vicious pathological cycle. Neurotransmitter imbalance impairs neuronal function, triggering microglial activation and neuroinflammation. Excessive inflammatory cytokines disrupt neurotransmitter homeostasis and induce mitochondrial dysfunction, leading to reactive oxygen species (ROS) accumulation, and oxidative stress further aggravates neuronal damage and inflammatory amplification. In turn, the above pathological microenvironment and characteristics induced by depression not only generate stimuli different from those in normal environments (such as ROS) but also exhibit special therapeutic targets, providing a feasible strategy and guidance for constructing functional DDNs for depression treatment. Briefly, nanosystems target these pathways via multi-dimensional strategies, including enzyme-mimetic activity compensation, non-coding RNA targeting, and neuroplasticity repair for neurotransmitter imbalance [14,15,16,17,18,19,20,21,22,23,24,25,26,27,28]; biomimetic design, inflammatory cytokine regulation, and epigenetic intervention for neuroinflammation [29,30,31,32,33,34,35,36,37,38,39,40,41,42,43,44]; and stimulus-responsive release, direct ROS scavenging, and crosstalk pathway blocking for oxidative stress [44,45,46,47,48,49,50,51,52,53,54,55,56,57,58,59,60,61,62,63,64,65,66,67,68,69]. Overall, functionalized nanodrugs, with their advantages of strong targeting, controllable drug release, high BBB penetration efficiency, and excellent biosafety, significantly overcome the limitations of traditional antidepressants, such as low permeability, significant side effects, and low treatment response rates. They provide diversified technical approaches and experimental evidence for the precise treatment of depression.

Despite the remarkable potential of functionalized nanodrugs for depression treatment, their translation from preclinical research to clinical practice remains impeded by multiple unresolved challenges, underscoring the need for focused advancements in future studies. First, enhancing brain region-specific targeting precision is paramount: current nanocarriers predominantly rely on broad-spectrum brain delivery, yet depression’s pathogenesis is tightly linked to discrete, functionally specialized brain regions (e.g., hippocampus, prefrontal cortex, amygdala). To address this, future designs should integrate brain region-specific receptor ligands (e.g., hippocampus-targeting RVG peptide, prefrontal cortex-specific TfR antibodies) or leverage neuron activity-dependent delivery mechanisms (e.g., responding to elevated Ca^2+^ levels in hyperactive depressive-related neural circuits) to achieve site-specific accumulation, ensuring that therapeutic payloads concentrate at pathological foci while minimizing off-target effects on healthy brain tissue. Second, strengthening multi-pathway synergistic intervention is critical, as depression’s pathology arises from the intricate crosstalk between neurotransmitter imbalance, neuroinflammation, and oxidative stress, forming a self-reinforcing vicious cycle. The development of “integrated” nanosystems that concurrently modulate multiple pathogenic nodes is therefore essential. For example, integrating ROS scavenging (e.g., enzyme-mimetic CeO_2_@BSA nanoclusters), inflammatory cytokine suppression (e.g., TDNs downregulating IL-1β/TNF-α), and neurotransmitter homeostasis restoration (e.g., Fe_3_O_4_@CS nanozymes reviving tryptophan hydroxylase function) can break this cycle, improve treatment response rates, and reduce the risk of disease chronicization. Complementing these strategies are proven nanotherapeutic approaches for individual pathways, such as biomimetic design (e.g., microglial cell membrane-wrapped PDA-Mem@M) and epigenetic intervention (e.g., HDAC inhibitor-loaded nanosystems activating autophagy) for neuroinflammation, or stimulus-responsive release (e.g., NIR light-controlled fasudil delivery) and Nrf2-ARE pathway activation (e.g., PB nanosystems) for oxidative stress—all of which underscore nanodrugs’ versatility in targeting depression’s complex pathology. Overall, these nanosystems, with their inherent advantages of enhanced targeting, controllable drug release, efficient blood–brain barrier penetration, and favorable biosafety, overcome the key limitations of traditional antidepressants (e.g., poor permeability, significant side effects, low treatment response rates) and provide diversified technical frameworks for precise depression treatment.

To accelerate the clinical translation of functionalized nanodrugs, addressing key translational aspects is indispensable, with a focus on pharmacokinetics, regulatory hurdles, large-scale production, and long-term neurotoxicity. In terms of pharmacokinetics (PK) and pharmacodynamics (PD), the unique physicochemical properties of nanomaterials (e.g., size, surface charge, biodegradability) necessitate tailored evaluation systems: non-invasive imaging techniques (e.g., PET-MRI, fluorescence molecular tomography) should be employed to track in vivo distribution, clearance, and metabolism in real time, while establishing PK/PD correlations in humanized models (e.g., humanized liver-kidney chimeric mice) to guide dosage optimization and avoid suboptimal efficacy or toxicity. Large-scale production is another critical bottleneck. Current laboratory-scale synthesis methods (e.g., batch reaction) lack scalability and consistency, highlighting the need for innovative manufacturing technologies such as microfluidic synthesis, continuous flow production, or 3D bioprinting—these approaches not only ensure uniform particle size, morphology, and drug-loading efficiency across batches but also reduce production costs, a prerequisite for widespread clinical accessibility. Long-term neurotoxicity evaluation is equally vital. Most preclinical studies focus on short-term efficacy (weeks to months), but nanomaterials’ potential bioaccumulation in the brain, immunogenicity, and impacts on neural plasticity or neurodevelopment require rigorous assessment using clinically relevant models (e.g., aged depression models, comorbid metabolic/neurodegenerative depression models, or non-human primates) with extended follow-up (6–12 months) to mimic long-term clinical use. Finally, navigating regulatory hurdles is essential. Regulatory agencies (e.g., FDA, EMA) require comprehensive data on nanomaterial safety, stability, and biocompatibility, necessitating standardized characterization protocols (e.g., ISO 10993 for biocompatibility [87]) and toxicological assessment frameworks tailored to nanodrugs. This includes demonstrating minimal systemic toxicity, no adverse effects on blood–brain barrier integrity, and a lack of long-term neuroinflammatory or neurodegenerative risks. By systematically addressing these translational challenges, functionalized nanodrugs can transition from preclinical promise to clinical reality, emerging as core tools for mechanism-based depression treatment and transforming mental health care from symptomatic management to targeted pathogenic intervention.

## 5. Conclusions

In sum, this review systematically summarizes the major pathogenic pathways of depression, as well as the mechanisms of action and research progress of functionalized DDNs in alleviating depression. The functionalized DDNs thus opened up a new path for the precise treatment of depression. With the deepening of basic research and the breakthrough of technical bottlenecks, they are expected to become a core means for overcoming depression in the future, providing solid theoretical and technical support for the development of precision medicine in mental diseases.

## Figures and Tables

**Table 2:** The construction principles for the representative functional DDNs and their therapeutic effects on depression.

Functionalized DDNs	Functional Strategies	Materials Type	Size/ζ-Potential	Target Ligand	Trigger Mechanism	Injection Route	Disease Model	BBB Penetration Metrics	Behavioral Outcomes/Efficacy	Limitation	Reference
Fe_3_O_4_@CS	CS modification and TPH-like nanozyme	Fe_3_O_4_	~50 nm/none	None	Tryptophan	Dose	Depression mouse model	2-fold Increase vs. Control	Stable like normal mice	Non-targeted and non-degradable	[61]
BP-RVG29 @HYP	RVG29 conjugation and photothermal effect	Black phosphorus nanosheets	~161 nm/−20 mV	RVG29 peptide	NIR	Tail vein	CUMS mouse model	~7.6-fold Increase vs. Control	Recovery to normal mice	Non-degradable	[12]
RVG29-Exo-133b	RVG29 Modification and Mir-133B loading	Exosomes	~180 nm/−3 mV	RVG29 peptide	Membrane fusion	Tail vein	PD-depression model mouse	~9-fold Increase vs. Control	Most significant Reduction In FST	Large-scale purification of exosomes	[73]
CFs@DP	NIR and magnetic dual response and control release	CFs	~60 nm/25.4 mV	Magnetic field	NIR and Catecholamine-Induced complexation	Atomization	schizophrenia model mouse	None	Alleviation of depressive-like behaviors	Biostability	[74]
PDA-Mem@M	BV2 cell membrane-coating and Mem loading	PDA	163.5 nm/−54.3 mV	Microglial membrane	Low acidic signal	Tail vein	CRS mouse model	~2-fold Increase vs. Control	Reversion of depressive symptoms	Biosafety and biostability	[75]
CAR-M-UZPM	Macrophages modification	UCNP	~70 nm for UCNP@ZIF-8/none	Macrophages and CAR	NIR	Tail vein	inflammation-related depression model mouse	~10-fold Increase s. Control	Reversion of depressive-like behaviors	Large-scale production and mass control	[79]
CeO_2_@BSA	BSA-incubation strategy and ROS quenching	CeO_2_	~2 nm/none	None	None	Tail vein	CRS mouse model	BBB crossing ability	Recovery to normal mice	Non-targeted and non-degradable	[81]
Olz/RDPA	Chemical grafting and ROS scavenging	Olz/DP nanoparticles	163.5 nm/3.67 mV	CPPs R6	ROS	Nose	CUMS mouse model	~60% penetration rate	Recovery to normal mice	Mass control and preparation complex	[82]
PBGE	Exosome modification and enzyme-like catalysis	PB	~120 nm/−18 mV	macrophage-secreted exosomes	Low acidic signal	Tail vein	Inflammation-induced depression model	~52% penetration rate	Reversion of depressive-like behaviors	Immunogenicity risk and large-scale production	[86]

## Data Availability

No new data were created or analyzed in this study. Data sharing is not applicable to this article.

## Abbreviations

The following abbreviations are used in this manuscript:

DDNs	Drug delivery nanosystems
SSRIs	Selective serotonin reuptake inhibitors
NaSSA	Noradrenergic and specific serotonergic antidepressants
SNRIs	serotonin-norepinephrine reuptake inhibitors
5-HT	serotonin
DA	Dopamine
NE	Norepinephrine
BBB	Blood–brain barrier
ROS	Reactive oxygen species
BDNF	Brain-derived neurotrophic factor
TPH	Tryptophan hydroxylase
TH	tyrosine hydroxylase
LPS	Lipopolysaccharide
GSH	Glutathione
FST	Forced swim test
TST	Tail suspension test
SPT	Sucrose preference test
OFT	Open field test
MAO	Monoamine oxidase
SOD	Superoxide dismutase
GPx	Glutathione peroxidase
XO	Xanthine oxidase
iNOS	Nitric oxide synthase
SERT	5-HT transporter
DAT	DA transporter
CS	Chitosan
MRT	Mirtazapine
MSNs	Mesoporous silica
MDD	Major depressive disorder
FLX	Fluoxetine
TDNs	Tetrahedral DNA nanostructures
PD	Parkinson’s disease
PFC	Prefrontal cortex
HPC	Hippocampus
CUS	Chronic unpredictable stress
PDA	Polydopamine
Mem	Memantine
CRS	Chronic restraint stress
UCNP	Up-conversion nanoparticles
SALNPs	Synergistic amine lipid nanoparticles
CAR	Chimeric antigen receptor
NIR	Near-infrared
RVG29	Rabies virus glycoprotein-29
Olz	Olanzapine
CFs	Carbonized MIL-100 (Fe) frameworks
DP	Domperidone
AB	Ammonia borane
DEX	Dextran
NNT	Nucleotide transhydrogenase
HA	Hyaluronic acid
PACAP	Pituitary adenylate cyclase-activating polypeptide
E2	Estrogen
5-HTP	5-hydroxytryptophan
PB	Prussian blue
GEN	Teniposide
HO-1	Oxygenase-1
NQO1	NAD(P)H: quinone oxidoreductase 1
CUMS	Chronic unpredictable mild stress
CNS	Central nervous system
